# Impact of non-fumigant nematicides on reproduction and pathogenicity of *Meloidogyne enterolobii* and disease severity in tobacco

**DOI:** 10.2478/jofnem-2023-0025

**Published:** 2023-06-05

**Authors:** Md Shah Alam, Churamani Khanal, Joseph Roberts, William Rutter

**Affiliations:** Clemson University, Department of Plant and Environmental Sciences, 105 Collings St., Clemson, SC 29634; Clemson University, Department of Plant and Environmental Sciences, Pee Dee Research and Education, Florence, 2200 E Pocket Rd, Florence, SC 29506; USDA-ARS, US Vegetable Laboratory, 2700 Savannah Hwy., Charleston, SC 29449

**Keywords:** biological, *Burkholderia*, fluensulfone, fluopyram, management, *Meloidogyne enterolobii*, nematicide, non-fumigant, oxamyl, tobacco

## Abstract

*Meloidogyne enterolobii* is a highly aggressive quarantine pathogen which threatens the multibillion-dollar tobacco industry and is not manageable with the currently available management methods in tobacco. There is currently no known host plant resistance in tobacco and previous studies have shown that the lower level of the currently recommended rate of non-fumigant nematicides does not provide satisfactory management of *M. enterolobii*. The current study was conducted with the hypothesis that *M. enterolobii* can be better managed using a single soil application of the maximum allowed rate of non-fumigant nematicides. Treatments involved three non-fumigant chemical nematicides (oxamyl, fluopyram, and fluensulfone), a biological nematicide derived from Burkholderia, and a non-treated control. Fluensulfone significantly suppressed the nematode reproduction relative to the control, the suppression being 71% for eggs and 86% for the second stage juveniles (J2). Fluopyram also suppressed nematode reproduction, although this was statistically insignificant, with the suppression being 26% and 37% for eggs and J2, respectively. Oxamyl significantly suppressed J2 (80%), but not eggs (50%) in relation to the control. The most significant reduction of disease severity was achieved by the application of fluensulfone (64%), followed by oxamyl (54%) and fluopyram (48%). Except for fluensulfone, which significantly reduced the root biomass, none of the nematicides significantly impacted root and shoot biomass. The biological nematicide did not significantly affect nematode reproduction, pathogenicity, or disease severity. The results from the current study suggest that while the non-fumigant nematicides provided a good level of the nematode suppression, more research is needed to improve the efficacy of non-fumigant nematicides through employing better application methods or finding better chemistries.

The guava root-knot nematode (*Meloidogyne enterolobii* Yang and Eisenback, 1983) is a highly aggressive species of *Meloidogyne* that attacks a wide range of economically important crops. This nematode has been reported to severely infect guava and tobacco plants in Brazil (Gomes et al., 2008). According to Schwarz et al. (2020), *M. enterolobii* has been found in several tobacco producing fields in North Carolina, suggesting a growing threat to tobacco production. Because *M. enterolobii* is a quarantine species and is not manageable with currently available nematode management practices, several states in the US have imposed a quarantine on the movement of soil and planting materials from nematode-infested areas (Ye et al., 2021). Studies are needed to develop better nematode management programs, not only because *M. enterolobii* poses a threat to the 15-billion-dollar tobacco industry in the US, but also to protect the well-being of farmers who have been farming tobacco for generations as their only source of income.

The current nematode management methods in tobacco are limited to the use of fumigants. Although effective in lowering the initial soil population of nematodes, fumigants do not provide season-long protection (Khanal et al., 2021). Additionally, they are cumbersome to apply and pose adverse impacts for the environment and human health (Khanal and Desaeger, 2020). Because fumigants work against a large range of organisms in the soil, they are also detrimental to beneficial microbiomes that play essential roles in maintaining soil health. In a quest to find safer yet effective alternatives to fumigants, some industries have developed and marketed non-fumigant chemical and biological nematicides. Non-fumigant chemical and biological nematicides are less harmful to the environment, have little to no off-target effect, and are easier to apply. Commonly available non-fumigant chemical nematicides include oxamyl (Vydate® L, Corteva Agriscience, Indianapolis, IN), fluopyram (Velum® Prime, Bayer CropScience, Research Triangle Park, NC), and fluensulfone (Nimitz®, ADAMA Agricultural Solutions Ltd., Raleigh, NC), while some biological nematicides include *Burkholderia* metabolites (Majestene®, Marrone Bio Innovations, Davis, CA) and Melocon (Certis® USA L.L.C., Columbia, MD). The efficacy of non-fumigant nematicides has been assessed for several crops; however, their efficacy on tobacco has not been studied extensively. A recent study of ours, which employed a single soil application of the lower level of recommended rate of oxamyl, fluopyram, fluensulfone, and *Burkholderia* metabolites found up to 99% suppression of *M. enterolobii* reproduction by the chemical non-fumigants while the effects of biological nematicide were similar to that of non-treated control (Alam et al., 2022). Because initial population of *M. enterolobii* even at a very low level can be highly damaging by the end of crop growing season and the non-fumigants at the lower level of currently recommended rate did not completely suppress the nematode reproduction, we hypothesize that a higher rate of nematicides could be helpful in further suppressing the nematode reproduction, pathogenicity as well as the disease severity. Therefore, the main objective of the current study was to evaluate the efficacy of a higher rate of non-fumigant nematicides on reproduction and pathogenicity of *M. enterolobii* as well as the disease severity in tobacco.

## Materials and Methods

### Nematode inoculum preparation

Nematode eggs were extracted from pure cultures of *M. enterolobii* originated from South Carolina and maintained on a tomato (*Solanum lycopersicum* L., cv. Rutgers, Seedway, Hall, NY) in a growth room. Galled roots of 3–4 months old tomatoes were cut into 3–5 cm pieces and agitated in 0.6% NaOCl for 4 min to dislodge the nematode eggs (Hussey and Barker, 1973). The extracted eggs served as inoculum, and inoculation was conducted within two hours of extraction.

### Establishment of experiment

Experiments were established in a Biosafety Level 2 growth room to comply with the biosafety requirements. Plastic pots with a 15 cm top diameter were filled with 1.5 kg sandy loam soil steam sterilized for four cycles of 45 min at 123°C. A waiting period 24 hr was maintained between pot filling and planting of tobacco seedlings to allow the release of possible toxic gases formed during soil sterilization. The soil-filled pots were placed in secondary containment on a bench to avoid floor contamination. Each pot received an 8-week-old tobacco seedling (cultivar K346, Gold Leaf Seed Company, Hartsville, SC) with four to six true leaves, and each pot was inoculated with approximately 10,000 eggs of *M. enterolobii*. The aqueous suspension of inoculum was pipetted into three 0.5 cm diam. × 5 cm deep depressions arranged into a triangular pattern and 2 cm away from the crown region.

Treatments included a single drench application of the maximum allowed rates of three non-fumigant chemical nematicides (oxamyl, fluopyram, and fluensulfone) and one *Burkholderia*-derived biological nematicide plus a control that received nematode inoculum but not nematicide (Table 1). Nematicides were applied with a pipette four days after egg inoculation in depressions prepared in a similar way as described for egg inoculation. An exception with nematicide application was made for pots receiving fluensulfone, where tobacco seedlings were transplanted in egg inoculated soil a week after nematicide application because of possible phytotoxicity as stated on the label. Pots receiving nematicides were watered with 100 mL water a few minutes before nematicide application and with 50 mL water after application to facilitate nematicide mobility in the soil. The experiment was established as a randomized block design with five replications of each treatment. A 12-hr photoperiod was provided by four metal halide bulbs (1,000 W) hanging approximately 3 m above the table. Standard watering, fertilization, and insect management practices were conducted. The entire experiment was repeated once. The average temperature for the first experiment was 27.5°C (maximum = 30.8°C, minimum = 20.6°C) with relative humidity of 45.6% (maximum = 48.3%, minimum = 43.7%). Similarly, the average temperature for the second experiment was 22.7°C (maximum = 29°C, minimum = 20.6°C) with a relative humidity of 47.2% (maximum = 50.1%, minimum = 45%).

**Table 1.** Non-fumigant and biological nematicides with their product application rates employed in the current study.

**Treatment**	**Application rate (L/ha)**	**Nematicide/pot (μl)**
Oxamyl	4.68	16.55
Fluopyram	1.00	0.06
Fluensulfone	8.18	0.48
^a^ *Burkholderia*	18.71	37.85
Untreated control	-	-

a *Burkholderia* rinojensis strain A396 secondary metabolites.

The experiments were terminated eight weeks after inoculation. Gall index (GI) data in a range of 0 -10 was collected for disease severity analysis (Bridge and Page 1980). Nematode eggs were extracted from whole root systems in each pot on the day of termination using the method of Hussey and Barker (1973) described above. Eggs were enumerated within 24 hr of extraction using a compound microscope (Martin Microscope Company, Easley, SC) at 40x magnification. Soil samples were placed in a walk-in cooler at 4°C, and second-stage juveniles (J2) were extracted from a 100 cm3 subsample of soil from each pot using the centrifugal flotation technique (Jenkins 1964). Soil extraction and enumeration of J2 were conducted within two weeks of experiment termination. The aboveground (shoot) and belowground (root) plant parts were dried in an incubator at 45°C for 2 weeks, and dry biomass was recorded to determine the pathogenicity of the nematode on tobacco.

### Data analysis

Nematode reproduction (eggs and J2) and pathogenicity (shoot and root biomass) data were subject to one-way analysis of variance (ANOVA) using the Generalized Linear Mixed Model in JMP PRO 16.2 (SAS Institute, Cary, NC). Residual analysis was performed to remove outliers. Subsequently, data were assessed for normality and any non-normal data were *log* transformed to fulfill the assumptions of ANOVA. Because of the absence of treatment by experiment interactions, data from two experiments were combined for analysis. Nematicide treatment was used as a fixed effect, and replication was used as a random effect. Tukey's HSD (*p* ≤ 0.05) was used for *post-hoc* mean comparisons. Untransformed values are presented in the Results section and figures.

## Results

### Effects of nematicides on nematode reproduction

**Figure 1:**
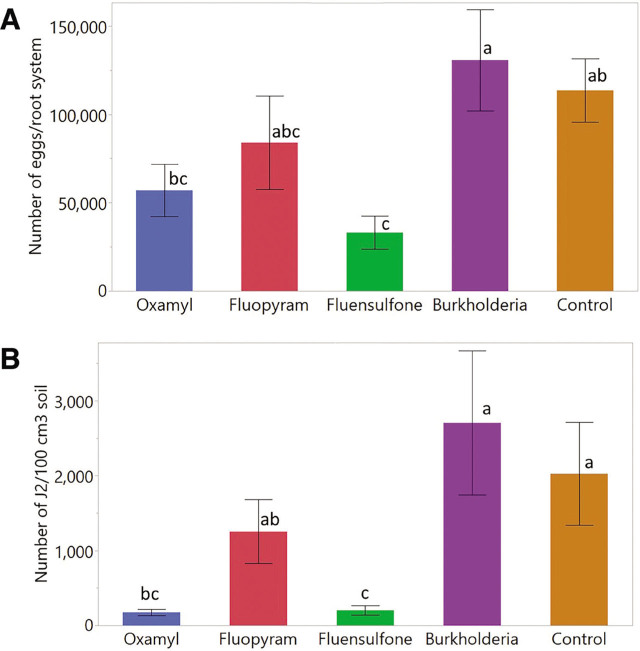
Reproduction of *M. enterolobii* expressed as number of eggs per root system of tobacco (A) and as number of J2 per 100 cm^3^ of soil (B). Data were combined over two experiments and are means of 10 replications. Data were log transformed and the treatment means followed by a common letter are not significantly different according to Tukey's HSD test (*P* ≤ 0.05). The treatment means in the figure represent untransformed values. *Burkholderia* refers to *Burkholderia rinojensis* strain A396 secondary metabolites.

Nematode reproduction was significantly impacted by the application of nematicides (*p* = 0.0005 for eggs and 0.0001 for J2). The effects of nematicides on nematode egg production are presented in Figure 1A. Across all treatments, the number of eggs/root system ranged from 2,280 to 320,400. Tobacco plants treated with fluensulfone had significantly lower nematode eggs relative to the control, the reduction being 71%. Oxamyl and fluopyram, respectively, suppressed 50% and 26% of nematode eggs; however, the suppressions were not significantly different from the control. The number of eggs in *Burkholderia* metabolites treated plants was statistically similar to those in the controls.

The effects of nematicides on the soil population of J2 are presented in Figure. 1B. The number of J2/100 cm^3^ soil across all treatments ranged from 10 to 8,100. Oxamyl and fluensulfone significantly suppressed the number of J2 relative to the control, the suppression being 88% and 86%, respectively. Fluopyram suppressed 37% of J2, although the suppression was not significantly different from the control.

### Effects of nematicides on nematode pathogenicity

**Figure 2:**
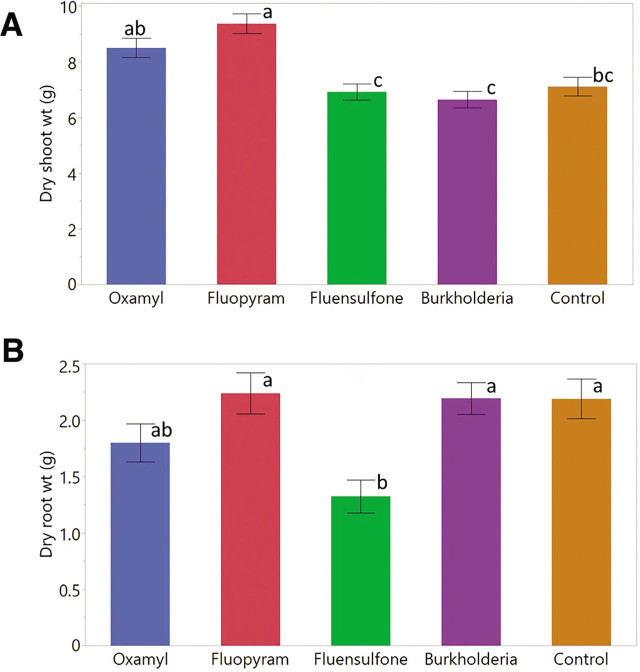
Dry weights of tobacco shoot (A) and root (B) inoculated with *M. enterolobii*. The plant materials were dried at 45°C for 2 wk. Data were combined over two experiments and are means of 10 replications. Treatment means followed by a common letter are not significantly different according to Tukey's HSD test (*P* ≤ 0.05). *Burkholderia* refers to *Burkholderia rinojensis* strain A396 secondary metabolites.

Nematicide application had a significant effect on plant shoot (*p* < 0.0001) and root biomass (*p* < 0.0001) as presented in Figures 2A and 2B. The dry shoot biomass across all treatments ranged from 4.5 to 11.3 g. Fluopyram treated plants had a significantly more, 27%, shoot biomass relative to the control. Although not significantly different, oxamyl and fluensulfone treated plants had 15% and 6% greater shoot biomass relative to the control. *Burkholderia* metabolites treated plants had 10% lower shoot biomass, although the reduction was not significantly different from the control.

The root dry biomass across all treatments ranged from 0.6 to 3.4 g. Only fluensulfone had a significant impact on the plant root biomass compared to the control. Fluensulfone treated plants had 39% less root biomass relative to the control. Fluopyram treated plants had 2% greater root biomass while oxamyl treated plants had 18% lower root biomass relative to the control. Plants treated with *Burkholderia* metabolites had slightly only 0.2% greater shoot biomass than the controls.

### Effects of nematicides on disease severity

**Figure 3:**
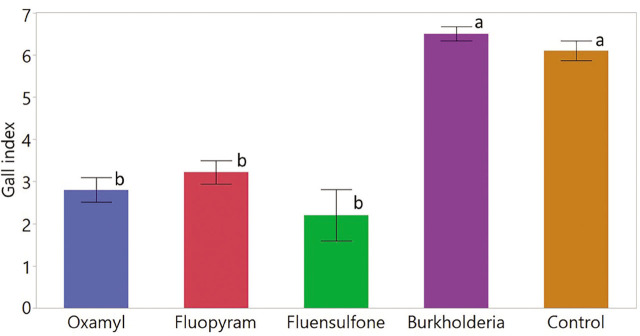
Root gall index as influenced by the application of nematicides. Gall rating was conducted on a scale of 0 -10 (0 = no galls, and 10 = completely galled roots) as described by Bridge and Page (1980). Data were combined over two experiments and are means of 10 replications. Treatment means followed by a common letter are not significantly different according to Tukey's HSD test (*P* ≤ 0.05). Burkholderia refers to *Burkholderia rinojensis* strain A396 secondary metabolites.

Nematode disease severity was significantly impacted by the application of nematicides (*p* < 0.0001) as presented in Figure 3. The GI ranged from 0 to 7 across all treatments. Plants treated with oxamyl, fluopyram and fluensulfone had significantly lower GI compared to that of controls. The greatest suppression of disease severity was achieved by the application of fluensulfone which suppressed 64% galls relative to the control. The suppression of disease severity by oxamyl and fluopyram was, respectively, 54% and 48%, relative to the control. The disease severity of plants treated with *Burkholderia* metabolites was statistically similar to that of controls.

## Discussion

*Meloidogyne enterolobii* has emerged as a significant constraint of modern agriculture. The current study evaluated the effects of non-fumigant chemical and biological nematicides on the reproduction and pathogenicity of *M. enterolobii* in tobacco. Although not always significant, a single application of the maximum allowed rate of the non-fumigant chemical nematicides was able to suppress nematode reproduction and pathogenicity. The *Burkholderia-*derived biological nematicide, however, was not able to suppress the nematode reproduction or pathogenicity. Nevertheless, results from the current study suggest that the non-fumigant chemical nematicides greatly suppress the reproduction and pathogenicity of *M. enterolobii* implying a possible alternative to fumigant nematicides in tobacco.

Research on the efficacy of non-fumigant nematicides against *M. enterolobii* in tobacco is limited. Our previous study was the first published report assessing the efficacy of non-fumigant nematicides against *M. enterolobii* in tobacco (Alam et al., 2022). The previous study employed a single soil application of the lower level of recommended rate of nematicides and found that oxamyl, fluensulfone, and fluopyram suppressed, respectively, 99.9%, 93%, and 99.9% of nematode eggs. Similarly, the suppression of J2 was 99% for oxamyl, 98% for fluensulfone, and 94% for fluopyram. The current study involving single soil application of the maximum allowed rate of nematicides was able to suppress up to 71% of eggs and 88% of J2 suggesting a greater efficacy was not achieved by increasing the amount of nematicide. Nevertheless, oxamyl and fluensulfone in the current study provided a significant suppression of nematode reproduction similar to those reported in our previous study. Fluopyram in the current study suppressed nematode reproduction; however, the suppression was not significantly different from the control. In contrast, our previous study found a significant suppression of nematode reproduction by the application of fluopyram. While it is unclear why the results were different, one possible reason could be the inoculum. The inoculum level in the current study was 10,000 eggs/pot while those in the previous study was 1,000 J2/pot. It is likely that J2 are more sensitive to nematicides than the eggs, which may have greatly reduced the initial population of J2. Another reason could be the timing of the nematicide application. Nematicides were applied two days after inoculation in the previous study while it was four days in the current study. Inoculating with eggs in the current study may have resulted in less exposure of J2 to the nematicides, due to variability in the timing of egg hatching and potentially differential susceptibility of unhatched nematodes. Further studies are needed to understand the interactions between the timing of J2 hatch and nematicide application timing in both greenhouse and field conditions.

Application of the non-fumigant chemical nematicides resulted in greater shoot biomass relative to the control, although not necessarily at a significant level, suggesting the pathogenicity of nematodes was reduced upon nematicide application. This finding is very useful for growers that are interested in moving away from the use of fumigants. The results from the current study align with the results from our previous study where the nematicides did not adversely affect the plant shoot weight (Alam et al., 2022). Interestingly, the plants treated with fluopyram in the current study had a significantly greater (27%) shoot biomass relative to the controls suggesting a higher dose of fluopyram possibly promotes shoot growth despite not being able to suppress the nematode reproduction significantly. Although the current study did not find adverse effects of the non-fumigant nematicides on shoot biomass quantity, further studies are needed to determine their effects on the quality of tobacco shoot biomass.

The root biomass of tobacco was significantly reduced by the application of fluensulfone while it was statistically unaffected by oxamyl and fluopyram implying the phytotoxicity of fluensulfone as indicated in the label. Our previous study also reported a significant reduction of root biomass in tobacco plants treated with fluensulfone (Alam et al., 2022). Oxamyl in the current study also reduced the root biomass, but the reduction was not significant. This result is in accordance with the previous study, which reported a non-significant suppression of root biomass by oxamyl. Although the root biomass was reduced upon application of oxamyl and fluensulfone, both nematicides significantly suppressed the nematode reproduction suggesting a trade-off between nematode suppression and host root biomass. Similarly, despite the inability to significantly suppress nematode reproduction by fluopyram, plants treated with this nematicide had a greater shoot and root biomass. Results from the current study suggest that the non-fumigant chemical nematicides are a viable option for managing *M. enterolobii* in tobacco.

Previous studies involving non-fumigant nematicides in other crops have reported inconsistencies in efficacies due to the nematode species, host crop, and environment. Oka and Saroya (2019) evaluated the *in-vitro* sensitivity of fluopyram and fluensulfone to *M. incognita* and *M. javanica* and found that the former was more sensitive to fluensulfone while the latter was more sensitive to fluensulfone. Fluopyram was effective in suppressing ring nematode in an in-vitro study (Kabir et al., 2021) while a field study did not find a suppressive effect of this nematicide on plant-parasitic nematodes (Schumacher et al., 2020). Ali and El-Ashry (2021) reported oxamyl to be effective against plant-parasitic nematodes while other studies reported it to be ineffective (Desaeger et al., 2004; Morris et al., 2015). A study by Khanal and Desaeger (2020) reported that the efficacy of non-fumigant nematicides depended on soil temperature, nematode species, the population density of nematodes, and crop type. An on-farm study found that fluopyram and oxamyl were able to suppress ring nematode populations in a peach field only for a few months citing their inability to provide season-long protection with a single application at currently recommended rates (Khanal et al., 2022). Looking into the results from previous studies, it has become apparent that the efficacy studies of non-fumigant nematicides conducted under controlled environments provide more consistent results than those conducted in the fields. Therefore, it is critical to conduct multiple on-farm efficacy studies to derive a more practical conclusion on the efficacy of nematicides. In most US states, however, on-farm studies on *M. enterolobii* are challenging because of the quarantine nature of the pathogen.

The *Burholderia* metabolite derived nematicide in the current study was not effective in suppressing the reproduction of *M. enterolobii*. Moreover, the reproduction of nematode on plants treated with *Burkholderia*-derived nematicide was higher than those in the controls, although not at a significant level. Although this nematicide did not have adverse effects on nematode reproduction, root and shoot biomass were not significantly impacted suggesting the other ingredients in the formulation may promote plant growth to compensate for the losses from the nematode attack. Several studies conducted in the past have reported the *Burkholderia*-derived nematicide to be ineffective in suppressing nematode reproduction (Watson and Desaeger, 2019; Alam et al., 2022; Khanal and Desaeger, 2020) suggesting a need for the development of biological nematicides with better efficacy.

The high levels of galling and reproduction of up to 320,400 eggs/root system supported by the tobacco cultivar K346 employed in the current study suggest the cultivar is susceptible to *M. enterolobii*. This reproduction is similar to the one reported by Khanal and Harshman (2020) involving 45-day experiments with 10,000 *M. enterolobii* eggs as initial inoculum on susceptible tomato cultivar Rutgers. In contrast, our previous study found a lower population of nematodes and indicated the possibility of the tobacco cultivar K346 carrying resistance against *M. enterolobii* (Alam et al., 2022). However, as explained earlier, the lower final population of nematodes may have resulted from the use of J2 as inoculum.

Non-fumigant nematicides employed in this study seem to be a promising tool to manage *M. enterolobii* in tobacco. Results suggest that further research is needed to improve the efficacy of non-fumigant nematicides. The means to increase the efficacy could be employment of better application methods such as multiple applications or finding better chemistries. Until host plant resistance becomes available, better management of *M. enterolobii* can be achieved by combining non-fumigant nematicides with other compatible management options such as crop rotation and cover cropping (Khanal and Harshman, 2020).
